# In Silico Exploration of Aryl Halides Analogues as Checkpoint Kinase 1 Inhibitors by Using 3D QSAR, Molecular Docking Study, and ADMET Screening

**DOI:** 10.15171/apb.2019.011

**Published:** 2019-02-21

**Authors:** Adib Ghaleb, Adnane Aouidate, Mohammed Bouachrine, Tahar Lakhlifi, Abdelouhid Sbai

**Affiliations:** ^1^Faculty of Science, Moulay Ismail University, Meknes, Morocco.; ^2^EST, Moulay Ismail University, Meknes, Morocco.

**Keywords:** 3D-QSAR, Molecular-docking, In silico ADMET, Chk1 inhibitors, Aryl halides

## Abstract

***Purpose:*** In this review, a set of aryl halides analogs were identified as potent checkpoint kinase 1 (Chk1) inhibitors through a series of computer-aided drug design processes, to develop models with good predictive ability, highlight the important interactions between the ligand and the Chk1 receptor protein and determine properties of the new proposed drugs as Chk1 inhibitors agents.

***Methods:*** Three-dimensional quantitative structure–activity relationship (3D-QSAR) modeling, molecular docking and absorption, distribution, metabolism, excretion and toxicity (ADMET) approaches are used to determine structure activity relationship and confirm the stable conformation on the receptor pocket.

***Results:*** The statistical analysis results of comparative -molecular field analysis (CoMFA) and comparative molecular similarity indices analysis (CoMSIA) models that employed for a training set of 24 compounds gives reliable values of Q^2^ (0.70 and 0.94, respectively) and R^2^ (0.68 and 0.96, respectively).

***Conclusion:*** Computer–aided drug design tools used to develop models that possess good predictive ability, and to determine the stability of the observed and predicted molecules in the receptor pocket, also in silico of pharmacokinetic (ADMET) results shows good properties and bioavailability for these new proposed Chk1 inhibitors agents.

## Introduction

Quantitative structure–activity relationship (QSAR) methodology is an essential tool in modern medicinal chemistry try to relate the biological activity of a series of chemicals to their physicochemical and structural properties, relying on the concept that similar structures can have similar properties and when the differences between compounds are high, the correlation of their properties with activities becomes hard, whereas the correlations between highly similar molecules are easier.^1^ The applications of QSAR to molecular modeling and drug discovery has led to developed tools in computational chemistry field, and have been used to predict a large number of biological endpoints and shed light on the mechanism of action, whether it is toxicological or pharmacological.

This study carried out comparative -molecular field analysis (CoMFA) and comparative molecular similarity indices analysis (CoMSIA) to predict the activity of 24 aromatic halides compounds present cytotoxicity activities retrieved from literature,^2-4^ and propose new competent drugs.

To study the stability of predicted compounds in serine-threonine kinase has an important role in repairing DNA damage and prevents cells from entering mitosis where DNA damage exists (ChK1 receptor) as inhibitor agents,^5^ a surflex-docking was performed. Also we calculated total scoring (energy affinity) and defined the stable conformation of the ligands and its interactions in the receptor pocket (PDB entry code: **6FC8**). Moreover we performed an in silico study concerning the absorption, distribution, metabolism, excretion and toxicity (ADMET), which has created a unique interdisciplinary interface between medicinal chemist and clinicians. These crucial proprieties are usually used to finalize clinical success of a drug candidate, because it has been estimated that 50% of drugs fail as results of poor bioavailability.

For a molecule crossing a membrane through passive diffusion, reasonable permeability can be made using molecular properties, such as lipophilicity or hydrogen bonding. For many drugs, this first requires metabolism or biotransformation, takes place in the gut wall during uptake, but primarily in the liver. Now softwares are available for BBB penetration, human intestinal absorption (HIA), Caco^-2^ permeability, P-gp efflux, mutagenicity, human hepatotoxicity, oral bioavailability, carcinogenicity, develop mental toxicity, metabolism, skin sensitization, substrates and inhibitors, CYP inducers, and PBPK.^6^

## Materials and Methods

A database of 24 compounds consisted of aryl halides analogs, the data set was split into two sets, 19 compounds were selected as training set and 5 compounds were selected as test set, based on a random selection to evaluate the ability of the model obtained. The structures of both the training and test sets are given in Table 1, while experimental and predicted biological activities are presented in Table 2. These data sets were used to construct 3D-QSAR (CoMFA and CoMSIA) models and to analyses their physicochemical properties. MIC activity was measured previously in μM/mL, we converted them to pLC_50_ values as Log(1/LC_50_). The pLC_50_ values presented in Table 2 were used as the dependent variables in all subsequently developed partial least squares (PLS) models.

**Table 1:** List of 24 halogen containing hydroxy and amino substituted aromatic compounds

**Comp**	** X**	** Y**	**R1**	**R2**	**R3**	**R4**	**R5**	** R6**	** R7**	** R8**
1	C	C	H	OH	CH_3_CO	H	Br	H	-	-
2	C	C	Br	OH	CH_3_CO	H	H	H	-	-
3	C	C	Br	OH	CH_3_CO	H	Br	OH	-	-
4	C	C	Br	OH	CH_3_CO	OH	Br	H	-	-
5	C	C	Br	H	CH_3_CO	H	Br	OH	-	-
6	C	C	Br	H	CH_2_ClCO	H	Br	OH	-	-
7	C	C	Br	H	Cl	H	Cl	OH	-	-
8*	C	C	Br	H	CH_3_CO	H	Br	NH_2_	-	-
9*	C	C	Br	H	CH_2_ClCO	H	Br	NH_2_	-	-
10*	C	C	Br	H	Cl	H	Br	NH_2_	-	-
11	C	C	H	Br	OH	Br	H	Br	-	-
12	C	C	H	Br	OH	Br	H	NO_2_	-	-
13	C	C	H	Br	OH	H	H	NO_2_	-	-
14*	C	C	H	Br	NH_2_	Br	H	Br	-	-
15*	C	C	H	Br	NH_2_	Cl	H	Br	-	-
16	C	C	H	Br	NH_2_	Br	H	NO_2_	-	-
17	C	C	H	Br	NH_2_	Cl	H	NO_2_	-	-
18	C	N	-	OH	Br	H	Br	CH_3_	-	-
19	N	N	-	NH_2_	-	H	Br	H	-	-
20	C	N	-	NH_2_	Br	H	H	NH_2_	-	-
21	C	N	-	NH_2_	Cl	H	Cl	H	-	-
22	-	C	Cl	H	Cl	OH	H	H	H	H
23	-	N	-	H	H	H	Br	H	Cl	OH
24	-	N	-	H	Cl	H	Cl	H	H	OH

* Test set molecules.

**Table 2:** Experimental and predicted activities of 24 aryl halides derivatives

**No.**	**pLC**_50_	**CoMFA**		**CoMSIA**	
		**Predicted**	**Residuals**	**Predicted**	**Residuals**
1	1.24	0.940	0.3	0.919	0.321
2	1.25	1.125	0.125	1.101	0.149
3	0.39	0.662	-0.272	0.683	-0.293
4	0.49	0.743	-0.253	0.782	-0.292
5	0.73	0.947	-0.217	0.697	0.033
6	0.03	0.124	-0.094	0.109	-0.079
7	0.49	0.452	0.038	0.357	0.133
8*	1.1	0.890	0.21	0.83	0.27
9*	0.3	0.172	0.128	0.163	0.137
10*	0.48	0.526	-0.046	0.408	0.072
11	0.27	0.253	0.017	0.180	0.203
12	1.12	1.288	-0.16	1.225	-0.105
13	0.98	1.340	-0.36	0.969	0.011
14*	0.6	0.591	0.009	0.490	0.11
15*	0.83	0.661	0.169	0.619	0.211
16	1.74	1.487	0.253	1.522	0.218
17	1.79	1.555	0.235	1.650	0.14
18	1.01	1.100	-0.09	1.23	-0.22
19	0.26	0.40	-0.14	0.33	-0.07
20	0.54	0.6	-0.06	0.52	0.02
21	0.65	0.59	0.06	0.77	-0.12
22	2.37	2.216	0.154	2.293	0.077
23	1.61	1.756	-0.146	1.601	0.009
24	2.6	2.478	0.122	2.233	0.367

The three-dimensional structure building of molecules and the optimizations were performed using Sybyl 2.0 program package.^7^ Discovery Studio,^8^ and the program MOLCAD. ADMET properties are determined by Admetsar and pKCSM predictors.^9,10^

### Minimization and alignment

**Figure 1:**
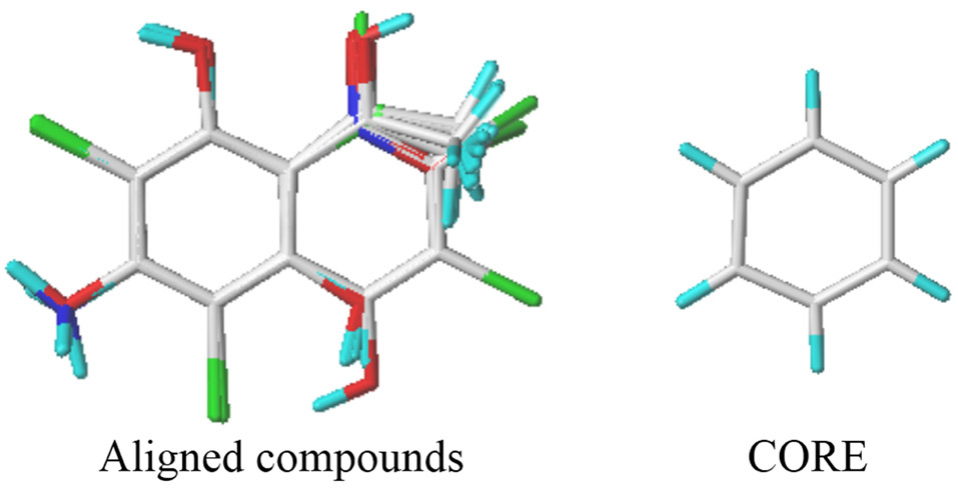
The superposition and alignment of training data set using compound **24** as a template.

All structures are sketched with SYBYL and optimized with Tripos force field,^11^ Gasteiger Huckel charges and with gradient convergence criteria 0.01 kcal/mol.^12^ The annealing simulation of structures is performed with 20 cycles. All molecules are aligned with common core, using simple alignment method,^13^ while active compound **24** is used as template. The superimposed structures are shown in Figure 1.

### 3D QSAR

Electrostatic, hydrophobic and steric fields contributions are explored to understand predicted activities, relying on CoMFA, CoMSIA studies and molecular alignment strategy, as previously described in literature.^14^

### CoMSIA and CoMFA

Relying on molecular alignment, CoMFA and CoMSIA approaches are applied to determine and analyze electrostatic, hydrophobic, and steric effects. Moreover, steric and electrostatic interaction fields are calculated at each intersection point of a regularly spaced grid of 2 Å, and default value of 30 kcal/mol is set as a maximum electrostatic and steric energy cutoff.^15^ Regression analysis used is the cross validation PLS method.^16^ The minimum column filtering is set to 2.0 kcal/mol, and the final non cross validated model is developed by optimal number of components with the highest Q^2^ value and the smallest value of standard error predictions. Moreover, the external validation R^2^ was used to evaluate power of predictive model. Many -CoMFA models were --built considering permutations of molecules- between training and test sets. The best model amongst them has been chosen, relying on high values of Q^2^ and R^2^, also with small standard error of estimate (SEE) value. The physicochemical properties for CoMSIA model have been adopted to avoid singularities at the atomic positions and dramatic changes of potential energy for grids being in the proximity of the surface. With the standard parameters and no arbitrary cutoff limits, five fields associated to five physicochemical properties were calculated.^17^

### PLS analysis

PLS method is a popular method used in deriving 3D-QSAR models,^18^ it is an extension of multiple regression analysis. In this rapport PLS method with leave-one-out (LOO) cross-validation is used to determine the optimal numbers of components relying on cross validation coefficient Q^2^. Moreover, an external validation is performed using a test set of five molecules. The final analysis is carried out to get correlation coefficient R^2^ and Q^2^ values, while Q^2^ determines the internal predictive ability of the model, and R^2^ value evaluates the internal consistency of the model.^19^ Thus, the best QSAR model is chosen relying on a combination of Q^2^ and R^2^.

### Y-Randomization test

The obtained models are validated by the Y-Randomization method.^20^ Y vector (-Log LC_50_) is randomly shuffled for many times and after every iteration. This technique is carried out to eliminate the possibility of the chance correlation, if obtained values of the Q^2^ and R^2^ are high, it means that 3D-QSAR model cannot be generated for this dataset because of structural redundancy and chance correlation.

### Molecular docking

Surflex-Dock module is used for molecular docking. The crystal structures of ChK1 kinase domain is downloaded from Protein Data Bank (PDB ID: **2FC8**).^21^ All water_ molecules in **2FC8** are deleted and the polar hydrogen atoms added. Protomol, is a computational representation of ligand that makes every potential interaction with binding site, applied to make molecular docking and predict the binding modes. All protomols can be established by three ways: (1) automatic: Surflex-Dock figure out the largest cavity in the protein; (2) ligand-based: ligand in the same coordinate space as the receptor; (3) residues based: this type specifying residues in protein,^22,23^ all ligands are docked in the pockets for further analysis.

An automatic docking is applied; the **2FC8** structure was utilized in the subsequent docking experiments without energy minimization with other parameters established by default in the software. Total scores are expressed in –log10(*K*_d_) units to present binding affinities. Then, Molecular Computer Aided Design program is employed to visualize binding modes and exhibits the surfaces of cavities.^24-26^ Moreover, Surflex-Dock scores, that represent binding energies, are used to determine ligand-receptor interactions of new predicted compounds. Each optimized conformation of every compound in the data set is energetically minimized using Tripos force field and Powell conjugated gradient algorithm with a convergence criterion of 0.05 kcal/mol and Gasteiger-Huckel charges.^12^

### In silico ADMET study

Drug discovery is a very complex and cost endeavor, divided into a series of stages including disease selection, target identification, lead or hit discovery, lead optimization, pre-clinical and clinical trials.^27^ In the past decade, many drug candidates have failed in late development stages. About 50% of drug failures can be attributed to unacceptable ADMET properties. This means that optimizing ADMET properties in early stage of drug discovery are widely used to reduce the high attrition rate. In recent years, a variety of medium to high-throughput in vitro ADMET screening methods have been developed. However, experimental evaluation of ADMET is still costly and time consuming. And still cannot meet the demands of drug screening and lead optimization. As the development of computer science and technology, in silico methods have been successfully applied to ADMET prediction.

In this review we focused on the development of ADMET prediction models for the excellent proposed compound.

## Discussion and Results

### CoMFA results

Results of Table 3 demonstrate that CoMFA model has high R^2^(0.94), F (13.56) values and a small S_cv_ (0.2) as well as cross validation coefficient Q^2^ (0.70) with forth as optimum number of components. The external predictive capability of QSAR model is cross checked and it is validated using test sets. The five randomly selected test sets are optimized and aligned in the same manner as training sets. The external validation gave high value of R^2^_ext_ (0.89) which indicates that prediction ability of CoMFA model is acceptable. The ration of steric to electrostatic contributions were found to be 60:40, which indicates that steric interactions are much more important than electrostatic.

**Table 3:** PLS Statistics of CoMFA and CoMSIA models

**Model**	**Q** ^ 2 ^	**R** ^ 2 ^	**S**_cv_	**F**	**N**	**r**_ext_^ 2 ^	**Fractions**				
							**Ster**	**Elec**	**Acc**	**Don**	**Hyd**
CoMFA	0.70	0.94	0.20	13.56	4	0.89	0.606	0.394	-	-	-
CoMSIA	0.68	0,85	0.33	49.62	3	0.96	0.079	0.122	0.297	0.00	0.502

R^2:^ Non-cross-validated correlation coefficient. Q^2:^ Cross-validated correlation coefficient. S_cv_: Standard error of the estimate. N: Optimum number of components. r_ext_^2^: External validation correlation coefficient. F: F-test value.

### CoMSIA results

3D-QSAR model was proposed based on CoMSIA descriptor to explain and predict quantitatively the hydrophobic, electrostatic, steric, donor and acceptor fields effects of substituents on anti-bacterial activity of 24 compounds. Different combinations of five fields were generated. The best proposed model of contains four fields (steric, electrostatic, hydrophobic, and acceptor), the correlation of cross-validated coefficient Q^2^ value of the training set and non-cross-validated correlation coefficient R^2^ of this model are 0.68 and 0.85, respectively. The optimal numbers of principal components used to generate the CoMSIA model are three which is reasonable, the standard error was 0.33.

**Figure 2:**
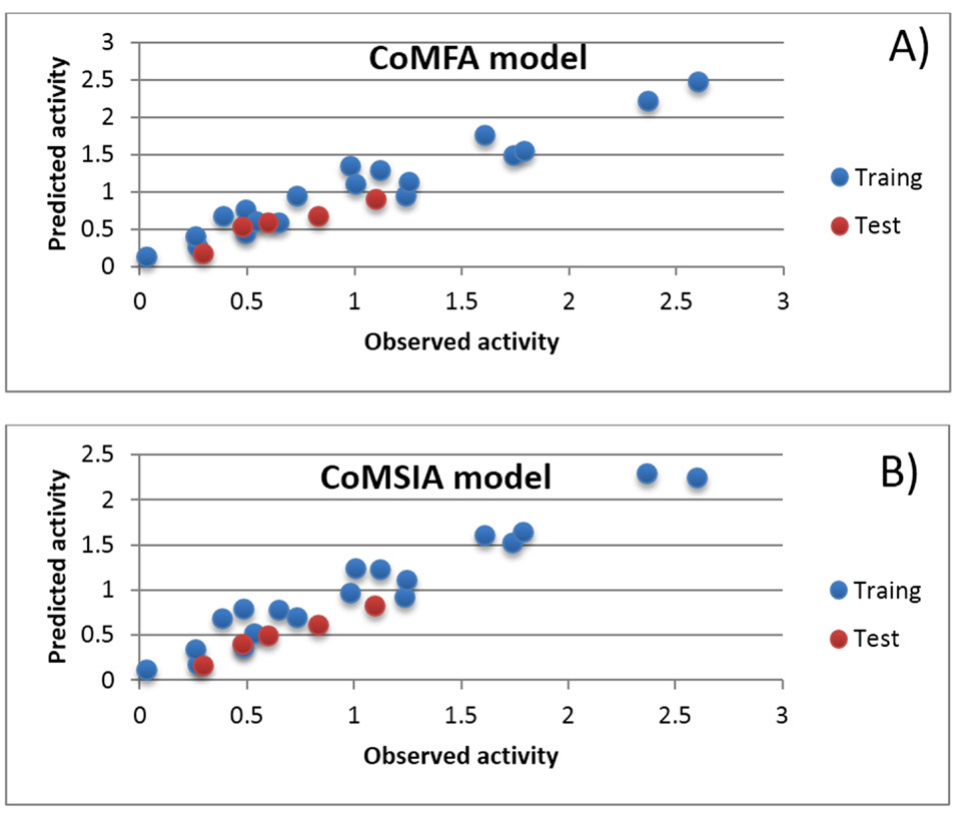
Plots of experimental and predicted pIC50 values for the total set in the CoMFA (A) and CoMSIA (B) models.

**Figure 3:**
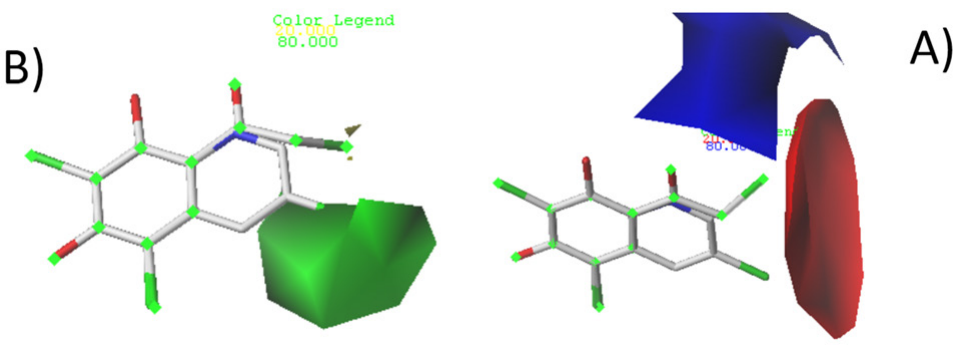
Contour maps of CoMFA with 2 Å grid spacing with compound 24. A) Electrostatic fields: blue contours show regions where groups with positive charges increase activity, while red contours indicate regions where groups with negative charges increase activity. B). Steric fields: green contours indicate regions where bulky groups increase activity, and yellow contours indicate regions where bulky groups decrease activity.

Finally, the prediction ability of the predicted model is confirmed by using an external validation (R^2^_ext_ value) that gave 0.96. These statistical results indicate good stability and the powerful predictive ability (Figure 2). Furthermore, the graphs showing the experimental and predicted pLC_50_ values for the total set used in the CoMFA and CoMSIA methods are described in Figure 3. The good linear relationships illustrated that the bioactivities predicted by the derived models are in agreement with the experimental data (see Figure 2), indicating that these models had satisfactory predictive capacity.

### Graphical interpretation of CoMFA and CoMSIA results

**Figure 4:**
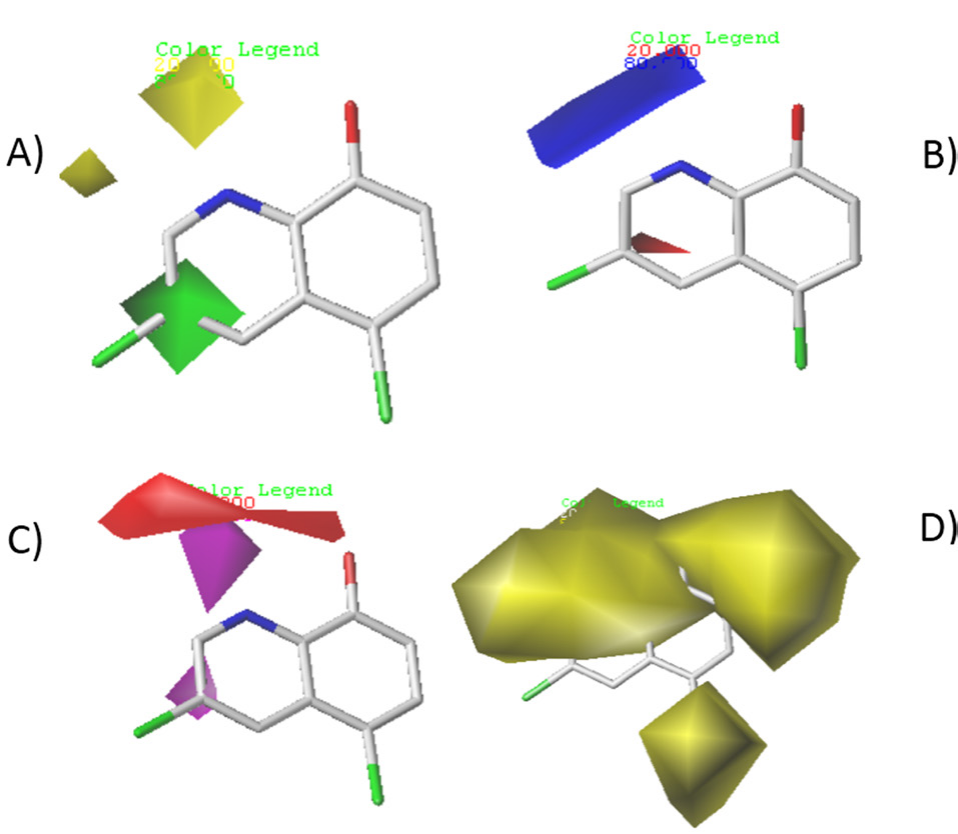
Contour maps of CoMSIA analysis with 2 Å grid spacing for compound **24**. A) Steric fields. B) Electrostatic fields. C) H-bond acceptor fields: D) Hydrophobic fields.

The contour maps of CoMSIA and CoMFA are generated to determine regions in 3D space around molecules where changes in each field are predicted either it can increase or decrease the activity. The steric and electrostatic contour maps of CoMFA are shown in Figure 3. While electrostatic, Steric, hydrogen bond acceptor and hydrophobic contour maps of CoMSIA are shown in Figure 4. Compound 4 and 24 are used as reference structures. All the contours represented default 80% and 20% level contributions for favored and disfavored regions, respectively.

### CoMFA contour maps

Electrostatic interactions in comparative molecular field analysis method are represented by red and blue contours while steric interactions are presented by green and yellow contours. Bulky substituents are favored in green regions, while at yellow regions, they are unfavored. Bleu regions indicate that positive charges are favored.

Figure 3 shows green and red contours around chlorine of quinoline compound (the active molecule), indicate that bulky groups with electron withdrawing character can increase the activity, the yellow and blue contours around dimethyl carbonyl of benzene compound (the inactive molecule), indicate that small groups with electron donating character are favored.

These results can explain why the compounds 22 and 24 with bulky and electron withdrawing groups around green and red contour maps present high activities, while compounds 6 and 9 with bulky and electron withdrawing around yellow and blue contour maps (R_3_= CH_2_ClCO) present low activities.

### CoMSIA contour map

The CoMSIA steric and electrostatic field contour maps indicate that bulky groups with electron withdrawing character are only favored at R_3_ position, the blue and yellow contours at R_1_ and R_2_ positions indicate that small groups with electron donating character are favored (see Figure 4). This is similar to CoMFA contour maps results.

The red contour around nitrogen and carbonyl groups indicates that groups with hydrogen bond donor character could increase the activity, while the purple contour at R_3_ position indicates that only groups with hydrogen bond acceptor character can increase the activity. Therefore, the hug yellow contour around the molecule shows the hydrophobic character of the active compound.

These results indicate that only hydrophobic compounds with bulky electron withdrawing groups at R_3_ position can increase the activity.

### Y-Randomization

The Y-Randomization method is carried out to validate CoMFA and CoMSIA models. Several random shuffles of the dependent variable were performed; after every shuffle, a 3D-QSAR was developed and the obtained results are shown in Table 4. The low Q^2^ and R^2^ values indicate that the good result in our original CoMFA and CoMSIA models are not due to chance correlation.

**Table 4:** Q^2^ and r^2^ values after several Y-randomization tests

**Iteration**	**CoMFA**		**CoMSIA**	
	**Q** ^ 2 ^	**r** ^ 2 ^	**Q** ^ 2 ^	**r** ^ 2 ^
1	-0.37	0.59	0.21	0.35
2	0.34	0.62	-0.07	0.22
3	-0.18	0.10	0.21	0.61
4	0.12	0.32	-0.19	0.02
5	0.14	0.26	0.05	0.28

### Design for new molecules with anti-obesity activity

Relying on CoMFA and CoMSIA models, new molecules have been designed to enhance the activity (Table 5). These compounds were aligned to database using compound **24** as a template.

**Table 5:** Chemical structure of newly designed molecules and their predicted pIC_50_ based on CoMFA and CoMSIA 3D-QSAR models

**No**	**Structure**								**Predicted pLC**_50_	
	**R**_1_	**R**_2_	**R**_3_	**R**_4_	**R**_5_	**R**_6_	**R**_7_	**R8**	**CoMFA**	**CoMSIA**
A1	-	O	CONH2	Me	Cl	Me	Me	OH	3.28	3.37
A2	-	O	OMe	Me	Cl	Me	Me	OH	2.98	2.26
A3	-	O	COMe	Me	Cl	Me	Me	OH	2.85	2.27
A4	-	O	CF3	Me	Cl	Me	Me	OH	2.29	2.58

The newly predicted structure A1 showed higher activity (pLC_50_ = 3.28 and 3.37 for CoMFA and CoMSIA models respectively) than compound **24** (the most active compound of the series pLC_50_ = 2.6).

Compounds with bulky electron withdrawing hydrophobic groups present high predicted activities, which means that the predicted compounds can be more effective that the compounds of the database.

To determine the stability and the interactions of these predicted molecules with the receptor, we applied surflex-docking.

### Docking results

**Figure 5:**
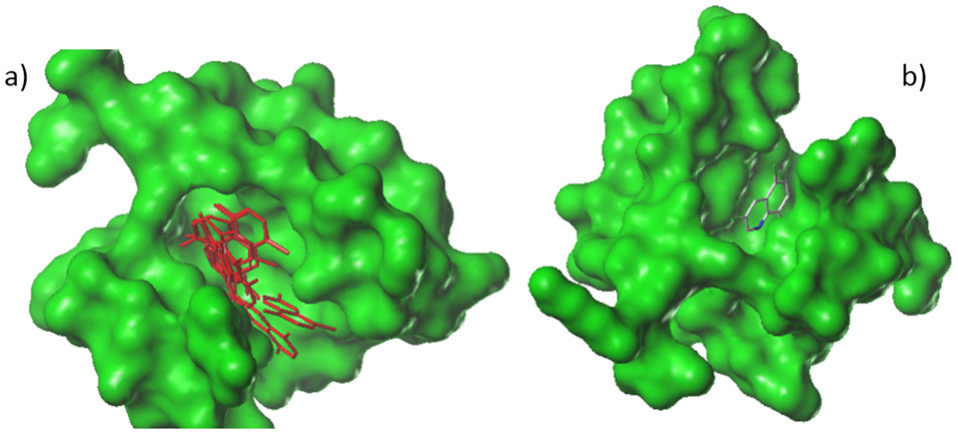
The MOLCAD surface of the site within the compound 24. a) different positions of compound 24 in the receptor. b) stable position of compound 24 in the receptor.

Molecular docking protocols are widely using to investigate the binding modes between the ligand derivatives and the receptor, which help understanding the 3D-QSAR study revealed by CoMFA/CoMSIA models. The target ligand taken from the crystal structure is redocked into the active site to validate the accuracy of molecular docking, the root mean square deviation (RMSD) value is 1.2 Å. Figure 5 shows the different top 10 positions of molecule 24 in the protein pocket, which present a stable conformation compared to compound 6 with scoring 3.7 and 1.2 respectively.

**Figure 6:**
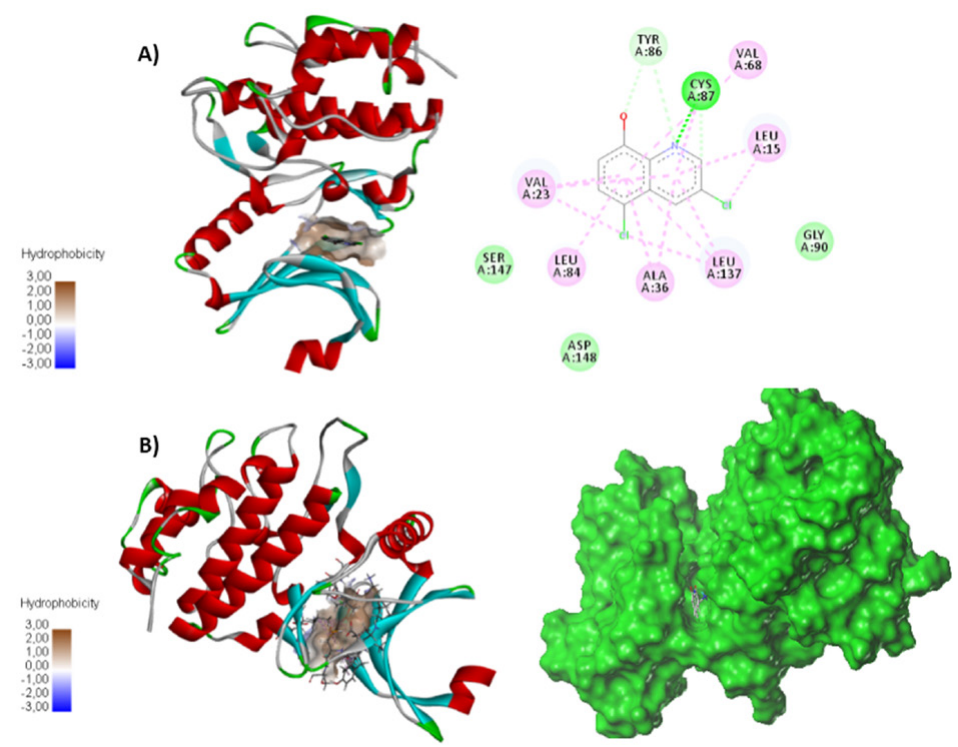
Docking interactions of compound 24 as the active compound in database.

Subsequently, the most active compound 24 was docked into the ligand-binding pocket of Chk1 protein (code PDB: 6FC8), as described in Figure 6 the docking results shows alkyl and pi-alkyl bonds with VAL23;VAL68; LEU84; LEU137; LEU15; ALA36 residues. The oxygen and nitrogen atoms present Van der Waals interaction with TYR86 residue, the nitrogen atom also form hydrogen-bond with CYS87 residue. These interactions can explain the stability of compound 24 in ChK1 protein pocket.

**Figure 7:**
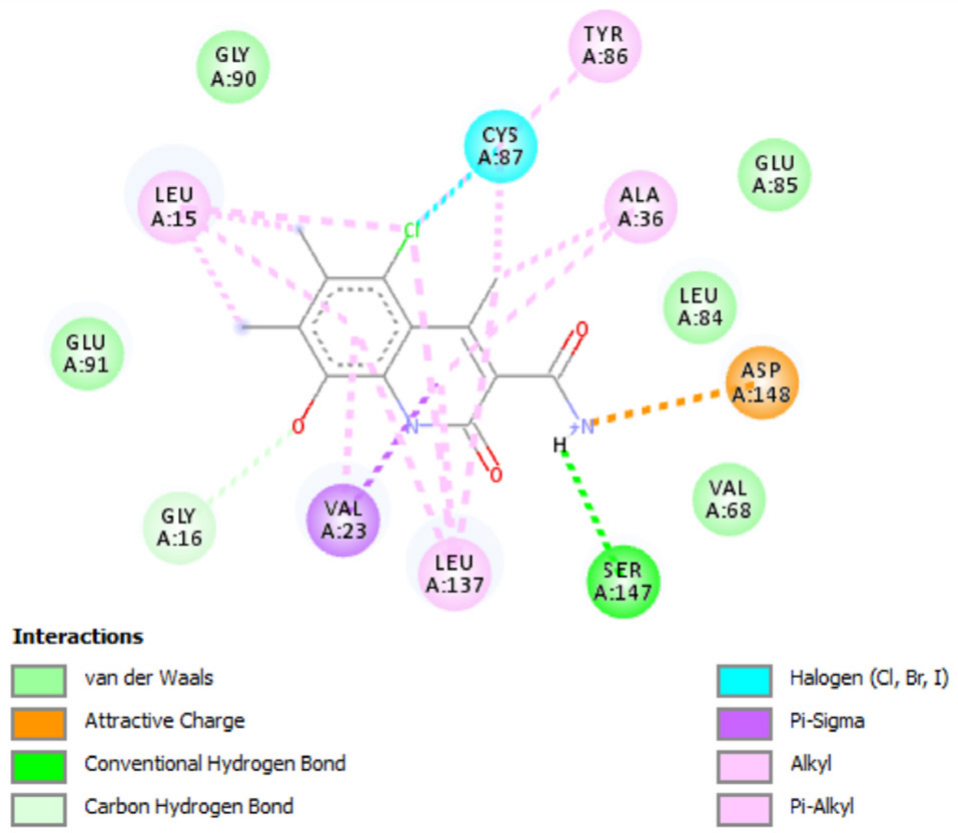
Docking interactions of the proposed compound A1.

Based on CoMFA/CoMSIA contour maps the hydrophobicity of compounds could increase the activity. Figure 7 shows that compound A1 (proposed compound with hydrophobic character) present Alkyl and pi-Alkyl bonds with LEU15; LEU137; ALA36; TYR86. In addition CYS87 residue presents halogen interaction with chlorine group, Van der Waals interaction between oxygen atom and VAL23 residue. The group amine –NH2 provided a hydrogen bond with SER147 residue and electrostatic interaction with ASP148 residue, which indicate that electro-withdrawing groups with hydrophobic character increase the stability of compound in the active protein pocket. Moreover, the results obtained by the docking had been compared with the OSAR results to verify mutually. These interactions match well with the results of H-bond acceptor/electrostatic contour maps.

### Drug-likeness or druggability

According to ‘Lipinski’s rule of five’, the lead compounds with poor absorption are known when there are more than 5 HBD, 10 HBA, the molecular weight higher than 500 Da and the calculated LogP (CLogP) is greater than 5.^28^ Moreover, an excellent bioavailability is likely for molecules with total polar surface area (TPSA) of ≤140 Å and rotatable bonds ≤10 (nrotb).^29^

In this study; in silico evaluation of new proposed Chk1 inhibitors drugs shows good oral bioavailability. The calculated LogP values, the number of hydrogen bond acceptors (HBA) and hydrogen bond donors (HBD) agreed with Lipinski’s rule of five. Furthermore, TPSA, total hydrogen bond count and the number of rotatable bonds felt within the limit ranges (see Table 6). Drug molecules of molecular weight less than 500 Da are easily transported, diffuse and absorbed as compared to heavy molecules.^30^ Which indicate that the proposed molecules present good bioavailability. Moreover, ADMET method applied to determine their properties and therapeutic benefit because Lack of efficacy and unacceptable toxicity of the new drug are mainly associated with failures of drug discovery.

**Table 6:** Physicochemical parameters of the three lead compounds

	**LogP**	**MW**	**TPSA**	**HBD**	**HBA**	**nrotb**
**Compound A1**	2.02126	278.695	113.553	2	3	1
**Compound A2**	3.01496	279.723	114.578	2	3	1
**Compound A3**	2.82096	267.712	109.165	2	3	1
**Compound A4**	3.83116	305.683	116.548	2	2	0

### ADME and toxicity results

During the drug discovery effort many drugs are miscarried due to blood brain permeation failure, toxicity and poor efficacy. The purpose of preclinical ADMET is to eliminate weak candidates. This allows drug-development resources to be focused on fewer but more-likely-to-succeed drug candidates. In this review will be treated the applicability of QSAR methods for the prediction of the ADMET profile of the new lead compounds as chk1 inhibitors anti-cancer agents. Therefore virtual properties were investigated, Absorption, distribution, metabolism, excretion, and toxicity (ADMET), which are key players in drug development.

The pharmacokinetic (ADMET) properties of the studied leads were calculated using admetSAR and pKCSM predictors. Blood-brain barrier (BBB) penetration, HIA, Caco^-2^ cell permeability and Ames test are used to refine the drug likeness properties.

The main interfaces separating the central nervous system (CNS) and the blood circulation are known as the BBB and the blood-cerebrospinal fluid barrier. BBB permeation is an important property for medicinal chemistry since it determines which drugs can or cannot pass the BBB and thereby exert its effect on the brain.^31^ For a given compound logBB<-1 considered poorly distributed to the brain. Therefore, BBB permeability results in Table 7 show non-penetrating (BBB^_^) for compounds A1 and A2 as new chk1 inhibitors drugs.

**Table 7:** Pharmacokinetic (ADMET) properties of the new anti-tuberculosis agents.

**Models**	**Compounds**			
	**Compound1**	**Compound2**	**Compound3**	**Compound4**
Absorption				
Blood-brain barrier (LogBB)	-0.241	-0.218	0.229	0.362
Intestinal absorption (human)	82.522	94.049	93.629	89.548
Caco^-2^ permeability	0.246	0.972	1.181	1.269
P-glycoprotein substrate	Yes	Yes	Yes	Yes
P-glycoprotein inhibitor	No	No	No	No
Renal OCT2 substrate	No	No	No	No
Distribution and Metabolism				
CYP2D6 substrate	No	No	No	No
CYP450 3A4 Substrate	No	No	No	No
CYP1A2 inhibitor	Yes	Yes	Yes	Yes
CYP 2C9 inhibitor	No	No	No	No
CYP2D6 inhibitor	Yes	No	No	No
CYP2C19 inhibitor	No	No	No	Yes
CYP3A4 inhibitor	No	No	No	No
Excretion and Toxicity				
Human ether-a-go-go-related gene inhibition	No	No	No	No
AMES toxicity	No	No	No	No
Carcinogens	Non-carcinogens	Non-carcinogens	Non-carcinogens	Non-carcinogens
Hepatotoxicity	No	No	No	No

It was also found that all tested compounds could be absorbed by the human intestine, but they cannot penetrate to Caco^-2^ (see Table 7). Nevertheless, the tested compounds proved to be potential substrates for P-glycoprotein (P-gp) which effluxes drugs and various compounds to undergo further metabolism and clearance resulting in therapeutic failure because the drug concentration would be lower than expected.^32^

Inhibition of OCTs and OATs can affect drug-drug interactions in cases where a drug that is cleared by a particular renal transporter is coadministered with another drug which inhibits the same transporter. The result can reduce renal clearance, and that is not the case of these new proposed drugs.

The inhibition of cytochrome P450 may cause drug-drug interactions in which coadministered drugs fail to be metabolized and accumulate to toxic levels.^33^ Notwithstanding, some of the cytochrome P450 isoforms might be inhibited by one or more of the predicted compounds. Fortunately, the new Checkpoint kinases inhibitors did not show any acute toxicity or mutagenic effect, respecting Ames test data.

## Conclusion

Several aromatic halides analogs were identified as potentially effective oral Chk1 inhibitors through a series of computer-aided drug design processes, such as 3D-QSAR modeling and molecular docking. CoMFA/CoMSIA models showed good internal and external validation abilities with interesting statistical capacity. Based on their contour maps new and potential molecules with high Chk1 inhibitory activities are proposed. Meanwhile, molecular docking process was established to study the possible binding modes of inhibitors at the active pocket of Chk1. Some key residues, such as Asp148, Cys87, Leu137, and Ser147 were found. Hydrogen bonding and electrostatic forces were predicted to be the key interactions that confer bioactivity. In addition in silico ADMET study showed good properties for the new proposed Chk1 inhibitors. Overall, these results indicate that the optimal CoMFA/CoMSIA models, molecular docking and ADMET properties can be used to predict novel Chk1 inhibitors and guide the discovery of new potential analogs.

## Ethical Issues

Not applicable.

## Conflict of Interest

The authors declare that there are no conflicts of interests.

## Acknowledgments

We are grateful to the “Association Marocaine des Chimistes Théoriciens” (AMCT) for its pertinent help concerning the programs.

## References

[1] Roy K, Kar S, Das RN. Understanding the Basics of QSAR for Applications in Pharmaceutical Sciences and Risk Assessment. Academic Press; 2015. p. 1-479.

[2] Cramer RD, Patterson DE, Bunce JD (1988). Comparative molecular field analysis (CoMFA) 1 Effect of shape on binding of steroids to carrier proteins. J Am Chem Soc.

[3] Klebe G, Abraham U, Mietzner T (1994). Molecular similarity indices in a comparative analysis (CoMSIA) of drug molecules to correlate and predict their biological activity. J Med Chem.

[4] Khan MAE, Ali MI, Hashem MA (2009). Polymer-supported Ammonium Dichlorobromide(I) Reagent Promoted Halogenation of Hydroxy and Amino Substituted Aromatic Compounds. J Bang Chem Soc.

[5] Carrassa L, Damia G (2011). Unleashing Chk1 in cancer therapy. Cell Cycle.

[6] Dearden JC (2007). In silico prediction of ADMET properties: how far have we come?. Expert Opin Drug Metab Toxicol.

[7] Sybyl-X Molecular Modeling Software Packages, Version 2.0. St. Louis, MO, USA: TRIPOS Associates, Inc; 2012.

[8] Dassault Systèmes BIOVIA, Discovery Studio Modeling Environment, Release 2017. San Diego: Dassault Systèmes; 2016.

[9] Cao D, Wang J, Zhou R, Li Y, Yu H, Hou T (2012). ADMET evaluation in drug discovery 11 PharmacoKinetics Knowledge Base (PKKB): a comprehensive database of pharmacokinetic and toxic properties for drugs. J Chem Inf Model.

[10] Pires DE, Blundell TL, Ascher DB (2015). pkCSM: predicting small-molecule pharmacokinetic and toxicity properties using graph-based signatures. J Med Chem.

[11] Clark M, Cramer RD, Van Opdenbosch N (1989). Validation of the general purpose tripos 52 force field. J Comput Chem.

[12] Purcell WP, Singer JA (1967). A brief review and table of semiempirical parameters used in the Hueckel molecular orbital method. J Chem Eng Data.

[13] Vyas VK, Bhatt HG, Patel PK, Jalu J, Chintha C, Gupta N (2013). CoMFA and CoMSIA studies on C-aryl glucoside SGLT2 inhibitors as potential anti-diabetic agents. SAR QSAR Environ Res.

[14] Ghaleb A, Aouidate A, Ghamali M, Sbai A, Bouachrine M, Lakhlifi T (2017). 3D-QSAR modeling and molecular docking studies on a series of 2,5 disubstituted 1,3,4-oxadiazoles. J Mol Struct.

[15] Stahle L, Wold S (1988). 6 multivariate data analysis and experimental design in biomedical research. Prog Med Chem.

[16] Bush BL, Nachbar RB (1993). Sample-distance partial least squares: PLS optimized for many variables, with application to CoMFA. J Comput Aided Mol Des.

[17] Viswanadhan VN, Ghose AK, Revankar GR, Robins RK (1989). Atomic physicochemical parameters for three dimensional structure directed quantitative structure-activity relationships 4 Additional parameters for hydrophobic and dispersive interactions and their application for an automated superposition of certain naturally occurring nucleoside antibiotics. J Chem Inf Comput Sci.

[18] Wold S (1991). Validation of QSAR’s. Quant Struct Act Relat.

[19] Baroni M, Clementi S, Cruciani G, Costantino G, Riganelli D, Oberrauch E (1992). Predictive ability of regression models Part II: Selection of the best predictive PLS model. J Chemom.

[20] Rucker C, Rucker G, Meringer M (2007). y-Randomization and its variants in QSPR/QSAR. J Chem Inf Model.

[21] Dang W, Muto Y, Inoue M, Kigawa T, Shirouzu M, Terada T, et al. Solution structure of the RRM_1 domain of NCL protein. Protein Data Bank; 2005.

[22] Jain AN (2003). Surflex: fully automatic flexible molecular docking using a molecular similarity-based search engine. J Med Chem.

[23] Sun J, Cai S, Mei H, Li J, Yan N, Wang Y (2010). Docking and 3D QSAR study of thiourea analogs as potent inhibitors of influenza virus neuraminidase. J Mol Model.

[24] Ai Y, Wang ST, Sun PH, Song FJ (2010). Molecular modeling studies of 4,5-dihydro-1H-pyrazolo[4,3-h] quinazoline derivatives as potent CDK2/Cyclin a inhibitors using 3D-QSAR and docking. Int J Mol Sci.

[25] Lan P, Chen WN, Chen WM (2011). Molecular modeling studies on imidazo [4, 5-b] pyridine derivatives as Aurora A kinase inhibitors using 3D-QSAR and docking approaches. Eur J Med Chem.

[26] Lan P, Chen WN, Xiao GK, Sun PH, Chen WM (2010). 3D-QSAR and docking studies on pyrazolo [4, 3-h] qinazoline-3-carboxamides as cyclin-dependent kinase 2 (CDK2) inhibitors. Bioorg Med Chem Lett.

[27] Caldwell GW, Yan Z, Tang W, Dasgupta M, Hasting B (2009). ADME optimization and toxicity assessment in early- and late-phase drug discovery. Curr Top Med Chem.

[28] Lipinski CA, Lombardo F, Dominy BW, Feeney PJ (2001). Experimental and computational approaches to estimate solubility and permeability in drug discovery and development settings. Adv Drug Deliv Rev.

[29] Veber DF, Johnson SR, Cheng HY, Smith BR, Ward KW, Kopple KD (2002). Molecular properties that influence the oral bioavailability of drug candidates. J Med Chem.

[30] Srimai V, Ramesh M, Satya Parameshwar K, Parthasarathy T (2013). Computer-aided design of selective Cytochrome P450 inhibitors and docking studies of alkyl resorcinol derivatives. Med Chem Res.

[31] Upadhyay RK (2014). Drug delivery systems, CNS protection, and the blood brain barrier. Biomed Res Int.

[32] Ankomah P, Levin BR (2012). Two-drug antimicrobial chemotherapy: a mathematical model and experiments with Mycobacterium marinum. PLoS Pathog.

[33] Lynch T, Price A (2007). The effect of cytochrome P450 metabolism on drug response, interactions, and adverse effects. Am Fam Physician.

